# Genetic Diversity Analysis of *Sapindus* in China and Extraction of a Core Germplasm Collection Using EST-SSR Markers

**DOI:** 10.3389/fpls.2022.857993

**Published:** 2022-05-24

**Authors:** Jiming Liu, Shilun Gao, Yuanyuan Xu, Mianzhi Wang, Jia Jun Ngiam, Nicholas Cho Rui Wen, Joan Jong Jing Yi, Xuehuang Weng, Liming Jia, Jarkko Salojärvi

**Affiliations:** ^1^Key Laboratory for Silviculture and Conservation of the Ministry of Education, Beijing Forestry University, Beijing, China; ^2^National Energy R&D Center for Non-Food Biamass, Beijing Forestry University, Beijing, China; ^3^School of Biological Sciences, Nanyang Technological University, Singapore, Singapore; ^4^Yuanhua Forestry Biological Technology Co., Ltd., Sanming, China; ^5^Organismal and Evolutionary Biology Research Program, Faculty of Biological and Environmental Sciences, The Viikki Plant Science Centre, University of Helsinki, Helsinki, Finland

**Keywords:** genetic diversity, population structure, core germplasm, *Sapindus mukorossi*, *Sapindus delavayi*, *Sapindus rarak*, *Sapindus rarak* var

## Abstract

*Sapindus* is an important forest tree genus with utilization in biodiesel, biomedicine, and it harbors great potential for biochemical engineering applications. For advanced breeding of *Sapindus*, it is necessary to evaluate the genetic diversity and construct a rationally designed core germplasm collection. In this study, the genetic diversity and population structure of *Sapindus* were conducted with 18 expressed sequence tag-simple sequence repeat (EST-SSR) markers in order to establish a core germplasm collection from 161 *Sapindus* accessions. The population of *Sapindus* showed high genetic diversity and significant population structure. Interspecific genetic variation was significantly higher than intraspecific variation in the *Sapindus mukorossi*, *Sapindus delavayi*, and combined *Sapindus rarak* plus *Sapindus rarak* var. *velutinus* populations. *S. mukorossi* had abundant genetic variation and showed a specific pattern of geographical variation, whereas *S. delavayi*, *S. rarak*, and *S. rarak* var. *velutinus* showed less intraspecific variation. A core germplasm collection was created that contained 40% of genetic variation in the initial population, comprising 53 *S. mukorossi* and nine *S. delavayi* lineages, as well as single representatives of *S. rarak* and *S. rarak* var. *velutinus.* These results provide a germplasm basis and theoretical rationale for the efficient management, conservation, and utilization of *Sapindus*, as well as genetic resources for joint genomics research in the future.

## Highlights

- *Sapindus* is biodiesel, biomedical, and multifunctional economic forest species.
- Interspecific genetic variation was significantly higher than intraspecific variation in the *Sapindus* populations.
- *Sapindus mukorossi* showed a specific pattern of geographical variation, whereas *Sapindus delavayi*, *Sapindus rarak*, and *Sapindus rarak* var. *velutinus* showed less intraspecific genetic variation.
- A core germplasm collection was created that contained 40% of the initial population; it comprised 53 individuals of *Sapindus mukorossi*, nine of *Sapindus delavayi*, one of *Sapindus rarak*, and one of *Sapindus rarak* var. *velutinus*.

## Introduction

*Sapindus* is a widely distributed economic forest genus of Sapindaceae family; it is typically scattered as single plant or extremely small populations in temperate to tropical regions, with main biodiversity in Southeast Asia and America (Liu et al., 2017). Among *Sapindus*, *Sapindus mukorossi* Gaertn. (*S. mukorossi*), *Sapindus delavayi* (Franch.) Radlk. (*S*. *delavayi*), *Sapindus rarak* DC. (*S. rarak*), and *Sapindus rarak* var. *velutinus* (*S. rarak* var.) are concentrated in east and southeast Asia (Liu et al., 2017). Seed oils of *Sapindus* are suitable for the preparation of biodiesel under both American and European standards (D6751 and EN 14214, respectively) (Chakraborty and Baruah, 2013; Pelegrini et al., 2017; Caowen et al., 2019), owing to the high oil content (26.69–44.69%) and unsaturated fatty acid (mean: 86.21%) (Sun et al., 2017; Liu et al., 2021a). The pericarp of *Sapindus* also contains abundant triterpene saponins (4.14–27.04%) and sesquiterpenes (Xu et al., 2018; Liu et al., 2019). More than 70 of these triterpenoid saponin compounds have been identified (Xu et al., 2018) and shown to exhibit outstanding surface activity, antibacterial (Basu et al., 2015), elution (Mukhopadhyay et al., 2013; Mukherjee et al., 2015; Mukhopadhyay et al., 2016), pharmacological (Rodriguez-Hernández et al., 2015), and physiological properties (Singh and Singh, 2008). Saponins from the pericarp of *Sapinuds* are widely used in commercial soaps, shampoos, and body washes (Muntaha and Khan, 2015), seeds oils are utilized in biodiesel and premium lubricants, and seedlings are commonly used for landscaping in southern China. *Sapindus* is consequently recognized as a sustainable biodiesel, biomedical, biochemical, and multifunctional economic forestry species in China (Sun et al., 2016; Liu et al., 2017; Liu et al., 2021a) with annual production values exceeding 100M USD. However, with worldwide deforestation and the rapidly anthropogenic expanding, the habitat and populations of *Sapindus* have been severely damaged or vanished in recent centuries, and the genetic diversity of *Sapindus* faces unprecedented threats (Liu et al., 2017; Liu et al., 2021b). Hence, breeders have recently carried out several surveys and collections of *Sapindus* germplasm resources, and over 1,000 samples have been collected (Liu et al., 2017). However, due to inconsistencies in the timing, standards, and designation of germplasm collections, there is considerable homonymy, synonymy, and genetic redundancy within the resources. Therefore, a comprehensive evaluation of the genetic diversity in *Sapindus* and the construction of a rationally designed core germplasm collection are needed.

Germplasm resources form the foundation of forest genetic breeding, and the development of forest tree breeding and industry depends largely on the extent and diversity captured by these resources. However, redundancy in germplasm resources may lead to lower conservation and management efficiency. The construction of core germplasm collection is the optimal solution to genetic redundancy. Core germplasm collection is a subset of germplasm accessions that represents the minimum repeatability and maximum genetic diversity of one species (Frankel, 1984; Brown, 1989; Lv et al., 2020). They have been widely used for germplasm management, conservation, and application in crop, flower, and horticultural tree species. Most core germplasm collections represent only 5–20% of the total germplasm collected (Hintum et al., 2000; Lv et al., 2020), thereby reducing conservation and management costs and improving the efficiency of germplasm utilization. However, woody plant germplasm is predominantly derived from natural populations with brief history of domestication and long generation time, therefore the accessions have a high intrinsic genetic diversity and core germplasm collections typically represent 10–45% of the complete germplasm collections within these species (Belaj et al., 2012; Duan et al., 2017; Min et al., 2017; Preethi et al., 2020).

Molecular markers are one of the most powerful and inexpensive tools for analyzing genetic diversity and establishing core germplasm collections, compared to whole genome sequencing, resequencing, or transcriptome sequencing approaches. Microsatellite markers, also referred to as the simple sequence repeats (SSR) markers, have been widely applied in genetic breeding, variety identification, germplasm diversity evaluation and conservation (Powell et al., 1996). EST-SSR (expressed sequence tags microsatellite markers) markers not only have the beneficial characteristics of high intraspecific polymorphism, co-dominant nature, and high reproducibility, but also originate from genomic coding regions and thus directly reflect the diversity of the underlying genes (Adams et al., 1991; Wang et al., 2017; Parthiban et al., 2018). EST-SSRs have been commonly used to evaluate genetic diversity of *Dendrobium officinale* (Xie et al., 2020), *Paeonia rockii* (Guo et al., 2020), coconut (Preethi et al., 2020), and *Stevia rebaudiana* (Cosson et al., 2019) and to construct core germplasm collections of *Rosa roxburghii* (Min et al., 2017), crape myrtle (Ye et al., 2017), and olive (Dervishi et al., 2021). Previous studies have applied ISSR (inter-simple sequence repeat) and RAPD (random amplified polymorphic DNA) molecular markers to evaluate the genetic diversity of the *S. mukorossi* population (Mahar et al., 2011b; Diao et al., 2016), however, there have been no studies or reports on the construction of *Sapindus* core germplasm collection.

In this study, 18 EST-SSR markers were selected based on whole transcriptome sequencing and used to evaluate the genetic diversity and population structure of 161 *Sapindus* individuals. The aim was to obtain a representative core germplasm collection which would retain maximum amount of genetic diversity and population structure of the sampled *Sapindus* population. The core germplasm will enable more scientific and rational conservation, management, and utilization of the genetic resources in *Sapindus*.

## Materials and Methods

### Expressed Sequence Tag-Simple Sequence Repeat Marker Retrieval and Primer Design

Expressed sequence tag-simple sequence repeats were identified in transcript sequences from the *Sapindus* whole transcriptome sequencing project using MISA^1^ (Thiel et al., 2003), and Primer3 (Untergasser et al., 2012) was used to design EST-SSR primers. We screened all EST-SSR markers against eight *Sapindus* germplasm accessions representing different geographic origins and selected 18 pairs of highly polymorphic and stable EST-SSR markers (Table 1) (unpublished). The 18 EST-SSR primers were synthesized by Beijing Ruiboxingke Biotechnology Co. Ltd. and used in subsequent experiments.

**TABLE 1:** Information of 18 EST-SSR primers used in this study.

No	Abbreviation	Microsatellite marker	Multiplex	Repeat motifs	Forward primer sequence 5′ → 3′	Reverse primer sequence 5′ → 3′	Tm(°C)	Size range (bp)
1	S129	Samuk12G0105900	p3	(GAC)10	AGGAGATTCAAGTGGTGGCG	GACGACGTACACTGCTCCAT	59.83	180–216
2	S704	Samuk07G0120400	p3	(TGG)9	ACAACTGGCAAGAGATCGCA	CACACCTCCATTTGGCTCCT	59.96	216–240
3	S73	Samuk07G0117300	p2	(GA)11	TTTGGCAGGCCTGTTGATCA	ACGTGAGCAAGACCGACTTT	59.90	252–286
4	S36	Samuk03G0000600	p2	(AT)15	GTCACAGCTCAGGTGTTCCT	TCGCCACTCCTTTAGGCTTT	59.31	258–314
5	S78	Samuk07G0006800	p3	(CAA)11	GAAGCCGGATCTAATGGGCA	TCACTCCAACAGCCTTGTCC	59.89	174–198
6	S20	Samuk02G0314000	p2	(TA)10	CTTATCGGATGGCCCTGCTT	CGCACTCACGGTACACCTAA	59.76	212–250
7	S63	Samuk06G0002300	p2	(TA)11	TTGCTTTCTCGTTGGCCTCA	ACAGATTGTGGTTGGACGCA	60.18	252–272
8	S29	Samuk02G0156900	p2	(CT)12	TCAGCGTTGAAGAGCCACAG	AGTCTCTCAACGGTGCCATC	59.75	168–322
9	S140	Samuk14G0055000	p2	(TC)10	GCTACCCACAGCTCACAAGT	ACTCTGTGAGGAGGGTCAGA	59.22	212–222
10	S105	Samuk10G0092500	p3	(ATC)10	TTCTTCCGATTGAGCGCCAT	CGAATCCAGTGGCAGTAGCA	60.11	219–240
11	S714	Samuk07G0111400	p2	(TC)11	ATGGAAGTCGGCCTGTCAAG	ACAGAGCTACAGCACATGGG	59.75	286–332
12	S14	Samuk01G0267400	p2	(AT)11	CCAGTCTGAGGGCTGCATTT	AACAAGGGGGAGCTGTGATC	59.67	284–332
13	S449	Samuk04G0084900	p4	(AAAT)5	CTAGCTGTGGGGGCACATAC	GCATATTAGCACCGACCGGA	59.97	212–262
14	S143	Samuk14G0082300	p2	(CT)10	CTAAGCACTTGAGCCCAGCT	TACATCATGCGCGCTGAGAT	59.97	248–298
15	S13	Samuk01G0206300	p3	(TGA)9	CGGCACTGCTGTTTGAGTTC	CTGTCCACGCCACTGACATA	59.75	363–408
16	S543	Samuk05G0084300	p2	(AT)11	CGCTGCGTCTCTGTTTTTGT	ACTGGGGCAGATGAGTATGC	59.53	216–236
17	NG1	NewGene.10582	p2	(AT)12	CTCTTCGGCAGCAGGAATGA	GCTTTTTGTCGCCAGTCACA	59.62	248–274
18	NG2	NewGene.27440	p3	(AAG)11	TACAACGCATCCACAACCCA	ACTTTATGTGCCAGGCGTCT	59.68	258–285

*No., number; Tm, temperature of melting.*

### Plant Materials

**FIGURE 1:**
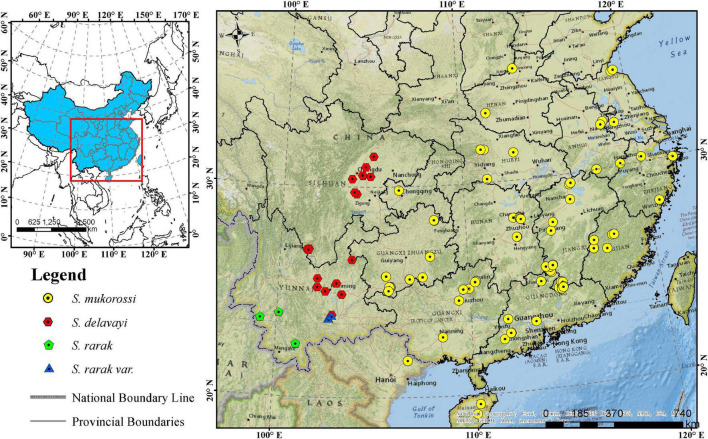
Geographic distribution of 161 *Sapindus* individuals. The map was generated using ArcMap 10.5 software.

The *Sapindus* population analyzed in this study comprised 161 wild individuals, with 160 individuals from 16 provinces in China and one individual from Vietnam (Figure 1 and Supplementary Table 1). It included 117 *S. mukorossi* individuals, as well as 36 *S. delavayi*, four *S. rarak*, and four *S. rarak* var. *velutinus* representatives. The criteria for *Sapindus* germplasm collection were representative local, naturally superior plants with a diameter at breast height of at least 30 cm. These germplasms were conserved *ex situ* by grafting at a *Sapindus* national germplasm nursery in Jianning County, Fujian Province, China (27°06′ N, 117°25′ E), comprising 175 clones and 64 half-sib families by grafting and seeding respectively, with at least 15 plants for each clone. The average temperature in germplasm nursery is 17.4°C, with a maximum temperature of 36.8°C (July) and a minimum temperature of 4.3°C (January), and an average relative humidity of 83.9% (Wang et al., 2020). At present, the grafted clones are 6 years old.

### DNA Extraction and Expressed Sequence Tag-Simple Sequence Repeat Genotyping

300 mg fresh leaf tissue from each *Sapindus* accession was used for DNA extraction with a Plant Genomic DNA Extraction Kit (Tiangen, DP320-03). The DNA samples concentration and quality were determined with a VERTEX 70 UV spectrophotometer (Bruker, Germany).

Eighteen EST-SSR markers were applied to genotype the 161 *Sapindus* tree samples. The forward primer of each marker was labeled at the 5′ end with a fluorescent dye (FAM, HEX, TAMRA, or ROX) according to the target fragment size of the marker. PCR (Polymerase Chain Reaction) was performed on a T100 thermal cycler (Biorad) in a 20-μL volume that contained 10.0 μL 2×Taq Plus PCR mix [Taq Plus DNA Polymerase (recombinant), 3 mM MgCl_2_, 0.4 mM dNTPs (dATP, dCTP, dGTP, and dTTP): 0.4 mM], 0.3 μL 10 μM forward primer, 0.3 μL 10 μM reverse primer, and 1.0 μL genomic DNA. The PCR protocol was 5 min denaturation at 94°C; 20 cycles of 30 s at 94°C, 30 s at 52/72/95/50°C (annealing temperature depended on the primer used, see Table 1), and 30 s at 72°C; and a final extension for 5 min at 72°C. Microsatellite alleles were called on an ABI 3730XL DNA analyzer, and the amplicons were statistically analyzed by GeneMarker 2.2.0 software (SoftGenetics, State College, PA, United States).

### Data Analysis

For each microsatellite marker, the number of alleles (Na), number of effective alleles (Ne), observed heterozygosity (Ho), expected heterozygosity (He), unbiased expected heterozygosity (uHe), ibreeding coefficient (F), and Shannon’s information index (I) were calculated using GenAlEx v6.5 (Peakall and Smouse, 2006). The polymorphic information content (PIC) was calculated using PowerMarker V3.25 software (Liu and Muse, 2005). *F*-statistics calculations (FIS, FIT, and FST) and principal coordinate analysis (PCoA) were also performed in GenAlEx v6.5 combined with Microsoft Excel. A neighbor-joining (NJ) tree was generated based on pairwise genetic distances between individuals by using PowerMarker V3.25 (Liu and Muse, 2005), and plotted with iTOL.^2^

The population structure analysis was performed with Bayesian model-based admixture analyses in STRUCTURE v2.3.4 (Pritchard et al., 2000). We set the number of Markov chain Monte Carlo (MCMC) iterations after burn-in to 100,000 with a 100,000-run length, and the number of genetically homogeneous clusters (*K* value) ranged from 1 to 20 with 10 replicate runs for each analysis. The optimum *K*-value was determined by the highest ΔK method (Evanno et al., 2005) in Structure Harvester^3^ (Earl and VonHoldt, 2012). The structure plot was constructed in R 4.1.0 (R Core Team, 2013).

The optimal set of core germplasm was extracted by the Core Hunter 3 (De Beukelaer et al., 2018) which maximized the genetic variation and allelic richness using local search algorithms. Based on the previously reported distribution of core germplasm fractions in woody plants ranging from 10 to 45% (Belaj et al., 2012; Duan et al., 2017; Feng et al., 2018; Lv et al., 2020), we decided to test 10 sampling fractions (10, 15, 20, 25, 30, 35, 40, 45, and 50% and initial group) respectively by Core Hunter 3. Na, Ne, Ho, He, I, and uHe were calculated separately for each fraction using GenAlEx software, as described above. These indicators were *t*-tested between the core subset and the initial group using Microsoft Excel. The smallest core subset that did not differ significantly with the 100% population group (*P* ≤ 0.05) was then selected as the optimal core germplasm collection (Lv et al., 2020).

## Results

### Genetic Diversity of *Sapindus*

There were 236 alleles identified by the 18 EST-SSR markers. All 161 individuals could be uniquely genotyped using these 236 alleles, demonstrating the high discrimination capacity of these 18 EST-SSR markers. The markers showed considerable variation (Table 2), with number of alleles (Na) ranging from 6 to 25 (mean 13.1), number of effective alleles (Ne) from 2.625 to 11.503 (mean 5.711), observed heterozygosity (Ho) from 0.277 to 1.000 (mean 0.558), expected heterozygosity (He) from 0.619 to 0.913 (mean 0.798), unbiased expected heterozygosity (uHe) from 0.621 to 0.916 (mean 0.801), Shannon’s information index (I) from 1.202 to 2.683 (mean 1.937), and polymorphic information content (PIC) from 0.561 to 0.907 (mean 0.775). The marker with the highest number of alleles was S36 (25), and the marker with the lowest was S140 (6). The observed heterozygosity of all markers was lower than the expected heterozygosity, with the exception of S105. All markers exhibited high polymorphism (PIC > 0.5; Table 2). The S36 marker captured the most genetic diversity with the highest PIC value (0.907).

**TABLE 2:** Genetic diversity parameters for *Sapindus* individuals at the 18 microsatellite markers.

No	Marker	*N*	Na	Ne	Ho	He	uHe	I	F	PIC
1	S129	159	11	5.226	0.277	0.809	0.811	1.864	0.658	0.783
2	S704	161	8	3.498	0.547	0.714	0.716	1.577	0.235	0.687
3	S73	159	14	6.353	0.623	0.843	0.845	2.114	0.261	0.826
4	S36	160	25	11.503	0.725	0.913	0.916	2.683	0.206	0.907
5	s78	160	9	5.023	0.419	0.801	0.803	1.771	0.477	0.772
6	S20	156	13	5.472	0.538	0.817	0.820	1.943	0.341	0.795
7	S63	124	11	5.424	0.355	0.816	0.819	1.896	0.565	0.792
8	S29	155	18	10.609	0.781	0.906	0.909	2.541	0.138	0.898
9	S140	161	6	2.625	0.429	0.619	0.621	1.202	0.308	0.561
10	S105	161	8	4.745	1.000	0.789	0.792	1.720	–0.267	0.759
11	S714	161	12	5.312	0.516	0.812	0.814	1.944	0.365	0.790
12	S14	161	21	8.128	0.863	0.877	0.880	2.433	0.016	0.866
13	S499	160	11	5.690	0.725	0.824	0.827	1.998	0.120	0.804
14	S143	159	20	5.055	0.447	0.802	0.805	2.048	0.443	0.782
15	S13	161	12	5.085	0.522	0.803	0.806	1.822	0.351	0.777
16	S543	153	11	2.875	0.320	0.652	0.654	1.496	0.509	0.627
17	NG1	160	16	6.581	0.556	0.848	0.851	2.159	0.344	0.833
18	NG2	160	10	3.596	0.406	0.722	0.724	1.661	0.437	0.693
Mean(± SD)		157.3(±8.6)	13.1(±5.1)	5.711(±2.355)	0.558(±0.196)	0.798(±0.0.078)	0.801(±0.368)	1.937(±0.368)	0.306(±0.217)	0.775(±0.088)

*No., number; N, number of individuals; Na, number of alleles; Ne, number of effective alleles; Ho, observed heterozygosity; He, expected heterozygosity; uHe, Unbiased Expected Heterozygosity = [2N/(2N−1)] × He; I, Shannon’s information index; F, inbreeding coefficient = (He − Ho)/He = 1 − (Ho/He); PIC, polymorphic information content; SD, standard deviation.*

There were marked differences in genetic diversity among the four different *Sapindus* taxa (Table 3). *S. mukorossi* exhibited the highest Na, Ne, I, Ho, He, and uHe values and the lowest *F* value compared with *S. delavayi*, *S. rarak*, and *S. rarak* var. *velutinus*. By contrast, *S. rarak* var. *velutinus* showed lower levels of genetic diversity, probably because of its narrow distribution area and smaller number of individuals. The mean pairwise *F*_*ST*_ coefficient between the four species of *Sapindus* was 0.154 (Table 4); highest differentiation was between *S. delavayi* and *S. rarak* (0.183) and lowest for *S. mukorossi* versus *S. delavayi* (0.122).

**TABLE 3:** Genetic diversity parameters for four different *Sapindus* species.

Pop	*N*	Na	Ne	I	Ho	He	uHe	F
SM	115.83	11.83	5.12	1.82	0.60	0.77	0.77	0.22
SD	33.67	7.44	3.25	1.32	0.47	0.61	0.62	0.25
SR	3.89	2.94	2.40	0.83	0.38	0.47	0.54	0.27
SRV	3.89	2.89	2.34	0.85	0.37	0.49	0.57	0.35

*SM, S. mukorossi; SD, S. delavayi; SR, S. rarak; SRV, S. rarak var.; N, number of individuals; Na, number of alleles; Ne, number of effective alleles; Ho, observed heterozygosity; He, expected heterozygosity; uHe, Unbiased Expected Heterozygosity = [2N/(2N − 1)] × He; I, Shannon’s information index; F, inbreeding coefficient = (He − Ho)/He = 1 − (Ho/He).*

**TABLE 4:** The pairwise *F*_*ST*_ comparison among four different *Sapindus* population.

Comparison	*F*_*ST*_
SM vs. SD	0.122
SM vs. SR	0.151
SD vs. SR	0.183
SM vs. SRV	0.156
SD vs. SRV	0.137
SR vs. SRV	0.174

*SM, S. mukorossi; SD, S. delavayi; SR, S. rarak; SRV, S. rarak var.*

### Genetic Structure of *Sapindus*

**FIGURE 2:**
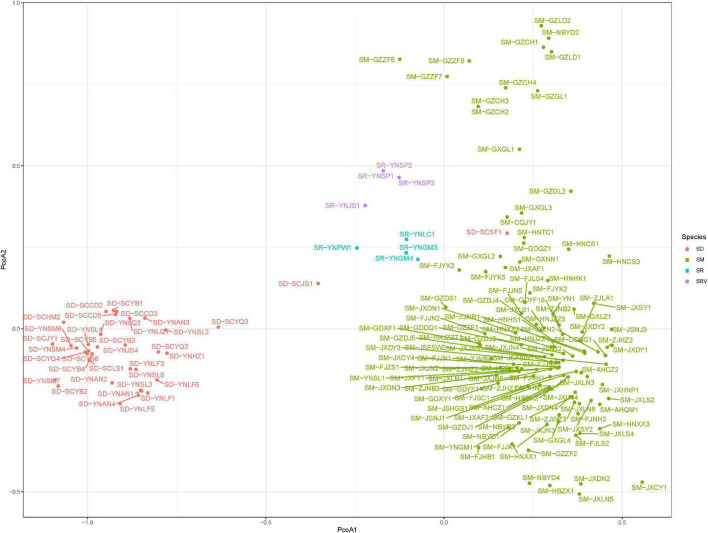
Principal coordinate analysis of 161 *Sapindus* individuals. SM, *S. mukorossi*; SD, *S. delavayi*; SR, *S. rarak*; SRV, *S. rarak* var. *velutinus.*

The first and second coordinates of the PCoA analysis accounted for 40.54 and 13.44% of the total genetic variation, respectively (Figure 2). The population of *Sapindus* was split into three clusters on the first principal coordinate axis (PCoA 1), which corresponded approximately to the *S. delavayi* group, the *S. rarak* and *S. rarak* var. *velutinus* group, and the *S. mukorossi* group. On the second principal coordinate axis (PCoA 2), individuals of *S. mukorossi* were roughly divided into two subgroups: the individuals from southern Guizhou province and the remaining *S. mukorossi* germplasm. Likewise, *S. rarak* and *S. rarak* var. *velutinus* were also divided into two subgroups along the second principal coordinate axis.

**FIGURE 3:**
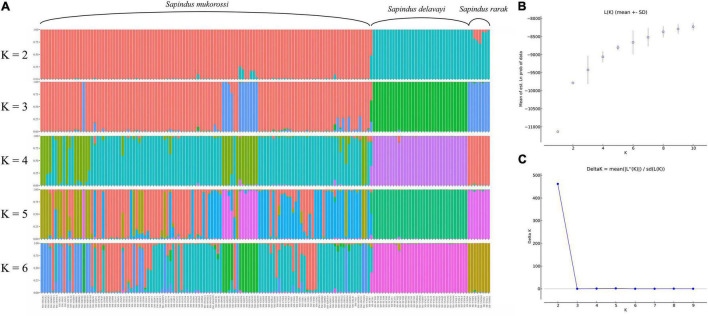
Structure analysis of 161 *Sapindus* individuals. **(A)** The population structure of *Sapindus* was determined using STRUCTURE 2.3.4 software (*K* = 2–6); **(B)** Estimated average likelihood L(*K*) distribution (mean ± SD) from 2 to 10 possible clusters (*K*); **(C)** Delta *K* distribution based on the rate of change in L(*K*) between continuous *K* values.

In contrast to the PCoA results, the STRUCTURE analysis indicated that the *Sapindus* population could be genetically divided into two distinct subgroups by the ΔK method (Figures 3B,C). Subgroup 1 contained all individuals of *S. mukorossi*, and subgroup 2 included all individuals of *S. delavayi*, *S. rarak*, and *S. rarak* var. *velutinus*. When K was equal to 3–6, individuals of subgroup 2 were consistently divided into *S. rarak* subgroup and *S. rarak* var. *velutinus* subgroup (Figure 3A). Furthermore, *S. mukorossi* individuals were divided into several subgroups (*K* = 3–6). Intriguingly, individuals from southern Guizhou province formed a separate subgroup (*K* = 3–6), suggesting that they originated from a distinct ancestral population.

**FIGURE 4:**
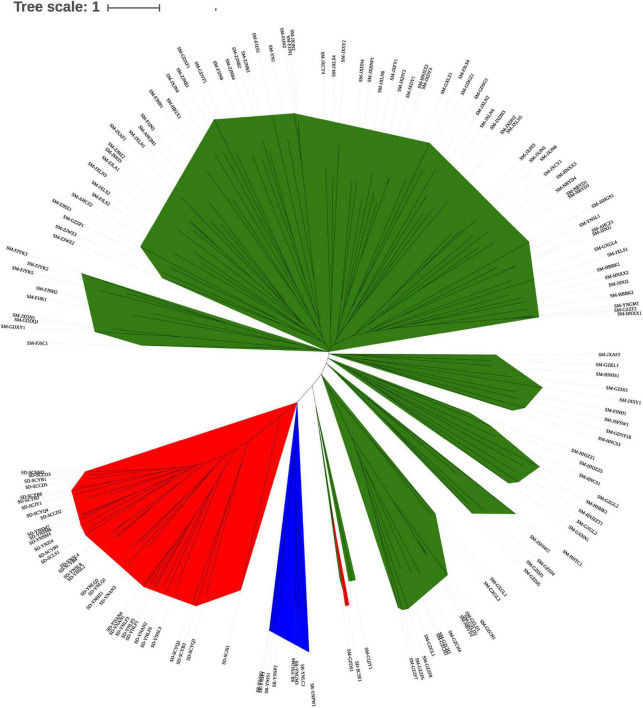
Dendrogram based on genetic distances among individuals in the *Sapindus* population. Green, *S. mukorossi*; yellow, *S. rarak* and *S. rarak* var. *velutinus*; red, *S. delavayi*.

The neighbor-joining dendrogram based on genetic distances among individuals (Figure 4) grouped *S. mukorossi*, *S. delavayi*, and *S. rarak* into their own populations, except for one *S. delavayi* individual that was grouped together with the *S. mukorossi* individuals. Furthermore, *S. mukorossi* individuals could be divided into multiple subgroups which largely correlated with their geographic distribution. Interestingly, some *S. mukorossi* individuals from Guizhou province appeared to be more closely related to *S. rarak.*

### Construction and Evaluation of a *Sapindus* Core Germplasm Collection

We produced nine candidate core germplasm collections of different sizes using Core Hunter. To determine the optimal core germplasm size, we compared the nine subgroups to the whole population according to six diversity parameters: Na, Ne, Ho, He, uHe, and I (Table 5). The core 10 and 15% subgroups differed significantly (*P* ≤ 0.05) and highly significantly (*P* ≤ 0.01) from the full population in Ne and I, respectively. The core 10–25% subgroups differed highly significantly (*P* ≤ 0.01) and significantly (*P* ≤ 0.05) in Na from the core 30 and 35% subgroups. The remaining parameters showed no significant differences between the subgroups and the full collection. Hence, the core 40% subgroup was selected as the optimal core germplasm collection. It contained 64 *Sapindus* individuals: 53 *S. mukorossi*, nine *S. delavayi*, one *S. rarak*, and one *S. rarak* var. *velutinus* (Supplementary Table 2), respectively. Relative to the full collection, the core 40% subgroup maintained 80.66% of Na, 97.65% of I, and 90.69% of Ho, and it had 101.90% higher Ne, 103.68% higher He, and 109.59% higher uHe.

**TABLE 5:** Comparison of genetic diversity parameters of different fractions of core germplasm subgroups.

	Fraction (%)	Na	Ne	I	Ho	He	uHe	F
Initial collection	100	7.76	3.89	1.41	0.48	0.64	0.66	0.29
Core 50%	50	6.53	3.92	1.37	0.43	0.66	0.71	0.37
Core 45%	45	6.33	3.97	1.38	0.44	0.67	0.72	0.37
Core 40%	40	6.26	3.97	1.38	0.43	0.67	0.72	0.38
Core 35%	35	5.88*	3.86	1.33	0.44	0.66	0.73	0.35
Core 30%	30	5.80*	3.89	1.34	0.44	0.66	0.73	0.36
Core 25%	25	5.64**	3.86	1.34	0.43	0.66	0.73	0.36
Core 20%	20	5.18**	3.59	1.26	0.42	0.64	0.72	0.36
Core 15%	15	4.47**	3.28*	1.08**	0.35	0.56	0.67	0.40
Core 10%	10	3.82**	2.99*	0.93**	0.37	0.48	0.59	0.21

Na, number of alleles; Ne, number of effective alleles; I, Shannon’s information index; Ho, observed heterozygosity; He, expected heterozygosity; uHe, Unbiased Expected Heterozygosity = [2N/(2N − 1)] * He; F, inbreeding coefficient = (He − Ho)/He = 1 − (Ho/He). *P ≤ 0.05 or **P ≤ 0.01 for difference between a core subset and the total population of Sapindus in simple t-tests.

## Discussion

### Genetic Diversity and Population Structure of *Sapindus* Germplasm

The collection of natural germplasm resources and genetic diversity evaluation is important for the conservation, breeding, and utilization of germplasm resources (Glaszmann et al., 2010). Previous studies have documented abundant genetic diversity in *Sapindus*. Diao et al. (2016) and Jiang et al. (2016) found significant genetic divergence among germplasm accessions of *S. mukorossi* using ISSR markers. Sun et al. (2018) also found significant interspecific genetic differences between *S. mukorossi* and *S. delavayi* in China using ISSR markers, and they reported that *S. mukorossi* could be broadly divided into two subgroups. In this study, for the first time, we have pooled *S. mukorossi*, *S. delavayi*, *S. rarak*, and *S. rarak* var. *velutinus* germplasms from China to analyze their genetic diversity and population structure using EST-SSR markers. We also found substantial genetic diversity within the *Sapindus* germplasm, with *S. mukorossi* exhibiting the highest genetic variation (Na = 11.83, I = 1.82); *S. rarak* and *S. rarak* var. *velutinus* showed less variation (Na = 2.94 and 2.89 and I = 0.83 and 0.85, respectively) (Tables 2, 3). This may be due to the wider distribution and population size of *S. mukorossi*; *S. rarak* and *S. rarak* var. *velutinus* have a limited distribution in Yunnan Province, China, with *S. rarak* found only in Xishuangbanna Dai Autonomous Prefecture, Yunnan Province and *S. rarak* var. *velutinus* only in Shiping and Jianshui counties in Honghe Hani and Yi Autonomous Prefecture, Yunnan Province.

It is difficult to distinguish *S. mukorossi*, *S. delavayi*, *S. rarak*, and *S. rarak* var. *velutinus* in nature because of their high phenotypic similarity, and there has been a lack of molecular biological support for the species splits in the *Sapindus* taxonomy. A better understanding of *Sapindus* genetic structure is a first step toward addressing these issues. Here, we have identified varying levels of genetic divergence among *S. mukorossi*, *S. delavayi*, *S. rarak*, and *S. rarak* var. *velutinus* for the first time using SSR markers. Pairwise *F*_*ST*_ comparisons among the four *Sapindus* taxa showed high genetic differentiation (Table 4), and PCoA (Figure 2) and a neighbor-joining dendrogram (Figure 4) supported this (Figure 2). However, structure analysis divided the *Sapindus* germplasms into two subgroups, a *S. mukorossi* subgroup and subgroup containing the other taxa (Figure 3A), probably due to the higher representation of *S. mukorossi* among the samples. Structure analysis was developed for comparing populations within a single species and requires the assumption of Hardy-Weinberg equilibrium in the population, which may be violated by the populations of *Sapindus* studied here (Lv et al., 2020). Hence, PCoA analysis can be a more valid and efficient approach for genetic structure identification owing to its relaxed Hardy-Weinberg equilibrium hypothesis (Lv et al., 2020). We presented the results of structure analysis for *K* = 3–6 and found that *S. mukorossi*, *S. delavayi*, and the combination of *S. rarak* and *S. rarak* var. *velutinus* were divided into three distinct subgroups; *S. mukorossi* could be further divided into multiple subgroups (Figure 3A). Intriguingly, structure analysis suggested that *S. rarak* and *S. rarak* var. *velutinus* originated from the same ancestral population (Figure 3A), and PCoA analysis also indicated that *S. rarak* and *S. rarak* var. *velutinus* were closely related (Figure 2), supporting the theory that *S. rarak* var. *velutinus* is a variety of *S. rarak*. Moreover, we also found that individuals from southern Guizhou Province formed a distinct subgroup (Figure 3A), consistent with the PCoA analysis in which *S. mukorossi* individuals were divided into two subgroups along PCoA axis 2 (Figure 2). Previously, (Mahar et al., 2011a,b, 2013) used RAPD, DAMD, and ISSR molecular markers to analyze germplasm of *S. mukorossi*, *Sapindus trifoliatus*, and *Sapindus emarginatus.* They found higher variation in genetic diversity within populations than between populations. Here, we found that *Sapindus* was genetically diverse, with interspecific genetic variation significantly higher than intraspecific variation. *S. mukorossi* had higher levels of genetic variation and showed a pattern of geographic variation, whereas *S. delavayi*, *S. rarak*, and *S. rarak* var. *velutinus* showed low levels of intraspecific genetic variation. These results differ from those of Mahar et al., perhaps because their population originated in India, where the germplasm collection was smaller and unevenly distributed (Mahar et al., 2011a,b, 2013). It is also possible that interspecific incompatibility is lower between the Indian species. Overall, we believe that these results provide molecular biological support for the current consensus taxonomy of *Sapindus* in China. Furthermore, it suggests that special attention should be paid to *Sapindus rarak* var. *velutinus* with respect to its relationship with the other *Sapindus* species when carrying phylogenetic studies in future.

### The Core Germplasm Collection of *Sapindus*

The construction of a core germplasm resource is an effective way to achieve efficient, scientific and rational conservation and utilization of genetic diversity (Xu et al., 2020), and the selection of an appropriate core population size is a crucial factor in establishing a core germplasm collection. Balakrishnan et al. (2000) and Zhang et al. (2010) suggested that the proportion of core germplasm should be determined by the size of the initial germplasm resource. Li et al. (2002) recommended sampling 5–40% of the core germplasm in crops, with 10% being optimal. However, woody plants are more genetically diverse, and the sampling percentage of core germplasm for woody plants is typically in the range of 10–45%. For instance, 14.71% (64/435) in apple (*Malus domestica* Borkh.) (Zhang et al., 2010), 17.96% (30/167) in *Citrus reticulata* (Garcia-Lor et al., 2017), 35% (247/707) in *Eucalyptus cloeziana* F. Muell (Lv et al., 2020), 35% (63/180) in *Ginkgo biloba* (Xuan et al., 2016), and 42.9% (300/700) in Chinese fir (Duan et al., 2017).

In this study, we selected a 40% (64/161) subgroup of the *Sapindus* core germplasm using Core Hunter. The population contained 53 *S. mukorossi*, nine *S. delavayi*, one *S. rarak*, and one *S. rarak* var. *velutinus* individuals (Supplementary Table 2). During the construction of core germplasm collections, allele retention is frequently considered as an evaluation criterion. For example, in *Saccharum officinarum* germplasm collection the criteria were to retain at least 70% of allele richness as well as other genetic diversity parameters (Balakrishnan et al., 2000). Compared with all *Sapindus* germplasm, the core germplasm collection had higher genetic diversity and maintained 80.66% of the allelic richness (Table 5), showing a balanced geographic composition (Supplementary Table 2). Previously, the core germplasm collection of lychee using 18 SSR markers resulted in 29.92% (38/127) individuals (Wang et al., 2012). The combined results indicate that the *Sapindus* core germplasm collection constructed in this study well represent the initial collection. The non-core germplasm of *Sapindus* population, also called reserve collection of *Sapindus*, is important for the conservation and utilization of *Sapindus* diversity, and it may harbor specific phenotypic, phenological and ecological characteristics to be of future use, thus it is important to conserve, exploit and understand the reserve collection as well. Although we have achieved our objectives of exploring genetic diversity and population structure in *Sapindus* and constructing a core germplasm collection using EST-SSR markers, our study still have some limitations. Compared with the millions of single nucleotide polymorphisms (SNP) and indel markers that can be obtained by whole genome resequencing or transcriptome sequencing, the number of EST-SSR markers in this study is relatively low, and the results obtained are not sufficient to fully elucidate the genetic structure of *Sapindus*. Hence, in future studies we hope to perform transcriptome sequencing or resequencing of this core germplasm collection to further explore interspecific and intraspecific genetic structure, evolutionary history, and regions under natural selection in *Sapindus*. Nevertheless, we believe the results of this study can help facilitate the efficient management, conservation, and utilization of *Sapindus* germplasm resources in the future.

## Conclusion

In this study, we have revealed high genetic diversity and significant genetic structure in *Sapindus* germplasms using 18 EST-SSR markers. Interspecific genetic variation was significantly higher than intraspecific variation in *S. mukorossi*, *S. delavayi*, and *S. rarak* plus *S. rarak* var. *velutinus* populations. *S. mukorossi* had abundant genetic variation and showed a pattern of geographic variation, whereas *S. delavayi*, *S. rarak*, and *S. rarak* var. *velutinus* showed less intraspecific genetic variation. A core germplasm collection was defined as 40% of the initial population; it comprised 53 *S. mukorossi*, nine *S. delavayi*, one *S. rarak*, and one *S. rarak* var. *velutinus*. The results obtained here provide a germplasm basis and theoretical rationale for the efficient management, conservation, and utilization of *Sapindus* germplasm, as well as genetic resources for joint genomics research in the future.

## Data Availability Statement

The datasets presented in this study can be found in online repositories. The names of the repository/repositories and accession number(s) can be found in the article/Supplementary Material.

## Author Contributions

JL, SG, LJ, and JS conceived and designed the project. YX completed the transcriptome sequencing of *Sapindus mukorossi*. JL, SG, MW, and XW collected the samples. JL, SG, and MW performed molecular labwork and scored the markers. JL, JN, NR, and JY analyzed the data. JL wrote the manuscript with input from JS, and feedback from all the authors. LJ contributed to the special foundation for National Science and Technology Basic Research Program of China and National Natural Science Foundation of China. JL contributed to the China Scholars Council. JS contributed to the Academy of Finland. All authors read and approved final manuscript.

## Conflict of Interest

XW was employed by Yuanhua Forestry Biological Technology Co., Ltd. The remaining authors declare that the research was conducted in the absence of any commercial or financial relationships that could be construed as a potential conflict of interest.

## Publisher’s Note

All claims expressed in this article are solely those of the authors and do not necessarily represent those of their affiliated organizations, or those of the publisher, the editors and the reviewers. Any product that may be evaluated in this article, or claim that may be made by its manufacturer, is not guaranteed or endorsed by the publisher.

## Footnotes

1
https://webblast.ipk-gatersleben.de/misa/

2
https://itol.embl.de/

3
http://taylor0.biology.ucla.edu/structureHarvester/
