# Supplementary material for: Genetic Diversity Analysis of Sapindus in China and Extraction of a Core Germplasm Collection Using EST-SSR Markers
Source: Front Plant Sci. 2022 May 24;13:857993. doi: 10.3389/fpls.2022.857993 (PMC9171133; doi:10.3389/fpls.2022.857993)
Supplement: Supplementary file 1 [file Data_Sheet_1.docx]

Table S1 Information of the origin of 161 *Sapindus* germplasm resources used in this study.

| Name | Location | Latitude | Longtitude | Species |  | Name | Location | Latitude | Longtitude | Species |
| --- | --- | --- | --- | --- | --- | --- | --- | --- | --- | --- |
| SM-HNXX1 | Xinxiang, Henan | 113.43 | 35.50 | S.M |  | SM-GZCH1 | Ceheng, Guizhou | 105.76 | 24.99 | S.M |
| SM-HNXX2 | Xinxiang, Henan | 113.43 | 35.50 | S.M |  | SM-GZKL1 | Kaili, Guizhou | 107.99 | 26.59 | S.M |
| SM-HNXX3 | Xixia, Henan | 111.58 | 33.41 | S.M |  | SM-GZDS1 | Dushan, Guizhou | 107.57 | 25.62 | S.M |
| SM-JSHGS1 | Lianyungang, Jiangsu | 119.29 | 34.64 | S.M |  | SM-FJLS2 | Shunchang, Fujian | 117.81 | 26.79 | S.M |
| SM-JSFSW1 | Lianyungang, Jiangsu | 119.43 | 34.71 | S.M |  | SM-FJLS4 | Shunchang, Fujian | 117.81 | 26.79 | S.M |
| SM-JSFSW2 | Lianyungang, Jiangsu | 119.43 | 34.71 | S.M |  | SM-FJYK2 | Shunchang, Fujian | 117.91 | 26.80 | S.M |
| SM-JSNJ1 | Nanjing, Jiangsu | 118.96 | 32.15 | S.M |  | SM-FJYK3 | Shunchang, Fujian | 117.91 | 26.80 | S.M |
| SM-JSNJ2 | Nanjing, Jiangsu | 118.16 | 32.16 | S.M |  | SM-FJYK5 | Shunchang, Fujian | 117.91 | 26.80 | S.M |
| SM-JSNJ3 | Nanjing, Jiangsu | 118.16 | 32.16 | S.M |  | SM-FJZS1 | Sanming, Fujian | 117.43 | 26.18 | S.M |
| SM-AHCZ1 | Chuzhou, Anhui | 118.26 | 32.25 | S.M |  | SM-FJNH1 | Ninghua, Fujian | 116.74 | 26.27 | S.M |
| SM-AHCZ2 | Chuzhou, Anhui | 118.29 | 32.29 | S.M |  | SM-FJNH2 | Ninghua, Fujian | 116.73 | 26.27 | S.M |
| SM-AHQM1 | Qimen, Anhui | 117.53 | 30.01 | S.M |  | SM-FJJN1 | Jianning, Fujian | 112.72 | 27.27 | S.M |
| SM-HBBK1 | Baokang, Hubei | 111.31 | 31.63 | S.M |  | SM-FJJN2 | Jianning, Fujian | 112.72 | 27.27 | S.M |
| SM-HBBK2 | Baokang, Hubei | 111.13 | 31.64 | S.M |  | SM-FJJK1 | Jianning, Fujian | 116.80 | 26.66 | S.M |
| SM-NBYD1 | Yidu, Hubei | 111.37 | 30.18 | S.M |  | SM-FJJN5 | Jianning, Fujian | 112.72 | 27.27 | S.M |
| SM-NBYD2 | Yidu, Hubei | 111.37 | 30.18 | S.M |  | SM-FJJN8 | Jianning, Fujian | 112.72 | 27.27 | S.M |
| SM-NBYD3 | Yidu, Hubei | 111.37 | 30.18 | S.M |  | SM-FJHB1 | Jianning, Fujian | 112.72 | 27.27 | S.M |
| SM-NBYD4 | Yidu, Hubei | 111.37 | 30.18 | S.M |  | SM-FJSC1 | Shunchang, Fujian | 116.80 | 26.66 | S.M |
| SM-HBZX1 | Zhongxiang, Hubei | 112.99 | 31.37 | S.M |  | SM-GDXY1 | Xinyi, Guangdong | 111.61 | 22.49 | S.M |
| SM-CQJY1 | Chongqin | 106.40 | 29.84 | S.M |  | SM-GDDQ1 | Deqin, Guangdong | 111.87 | 23.40 | S.M |
| SM-ZJWZ2 | Wenzhou, Zhejiang | 120.66 | 28.02 | S.M |  | SM-GDSG1 | Shaoguan, Guangdong | 113.75 | 25.04 | S.M |
| SM-ZJWZ3 | Wenzhou, Zhejiang | 120.66 | 28.02 | S.M |  | SM-GDYF18 | Yunfu, Guangdong | 111.98 | 22.75 | S.M |
| SM-ZJNB1 | Ningbo, Zhejiang | 121.86 | 29.96 | S.M |  | SM-GDYF1 | Yunfu, Guangdong | 111.95 | 22.75 | S.M |
| SM-ZJNB2 | Ningbo, Zhejiang | 121.86 | 29.96 | S.M |  | SM-GDXF1 | Yunfu, Guangdong | 111.96 | 39.86 | S.M |
| SM-ZJNB3 | Ningbo, Zhejiang | 121.86 | 29.96 | S.M |  | SM-GDGZ1 | Guangzhou, Guangdong | 113.29 | 23.18 | S.M |
| SM-ZJNB4 | Ningbo, Zhejiang | 121.86 | 29.96 | S.M |  | SM-GXGL1 | Guilin, Guangxi | 109.98 | 24.98 | S.M |
| SM-ZJLA1 | Hangzhou, Zhejiang | 118.90 | 30.11 | S.M |  | SM-GXGL2 | Guilin, Guangxi | 110.36 | 25.31 | S.M |
| SM-ZJHZ1 | Hangzhou, Zhejiang | 120.12 | 30.26 | S.M |  | SM-GXGL3 | Guilin, Guangxi | 110.31 | 25.28 | S.M |
| SM-ZJHZ2 | Hangzhou, Zhejiang | 120.12 | 30.26 | S.M |  | SM-GXGL4 | Guilin, Guangxi | 109.66 | 24.95 | S.M |
| SM-JXLS1 | Jiujiang, Jiangxi | 115.89 | 29.49 | S.M |  | SM-GXNN1 | Nanning, Guangxi | 108.49 | 22.75 | S.M |
| SM-JXLS2 | Jiujiang, Jiangxi | 115.89 | 29.49 | S.M |  | SM-GXLZ1 | Liuzhou, Guangxi | 109.41 | 24.44 | S.M |
| SM-JXLS4 | Jiujiang, Jiangxi | 115.98 | 29.55 | S.M |  | SM-HNHK1 | Haikou, Hainan | 110.19 | 19.57 | S.M |
| SM-JXJN2 | Nanchang, Jiangxi | 115.83 | 28.77 | S.M |  | SM-HNTC1 | Tunchang, Hainan | 110.01 | 19.12 | S.M |
| SM-JXJN3 | Nanchang, Jiangxi | 115.83 | 28.77 | S.M |  | SM-YN1 | Yuenan | 106.69 | 21.70 | S.M |
| SM-JXJN4 | Nanchang, Jiangxi | 115.83 | 28.77 | S.M |  | SD-SCSF1 | Jianyang, Sichuan | 104.82 | 30.50 | S.D |
| SM-JXJN5 | Nanchang, Jiangxi | 115.83 | 28.77 | S.M |  | SD-SCJS1 | Chengdu, Sichuan | 103.80 | 30.38 | S.D |
| SM-JXJN6 | Nanchang, Jiangxi | 115.83 | 28.77 | S.M |  | SD-SCYB1 | Yanbian, Sichuan | 101.50 | 26.92 | S.D |
| SM-JXAF1 | Anfu, Jiangxi | 114.58 | 27.39 | S.M |  | SD-SCYB2 | Yanbian, Sichuan | 101.51 | 26.92 | S.D |
| SM-JXAF2 | Anfu, Jiangxi | 114.58 | 27.39 | S.M |  | SD-SCYB3 | Yanbian, Sichuan | 101.50 | 26.91 | S.D |
| SM-JXFY1 | Fenyi, Jiangxi | 114.74 | 27.79 | S.M |  | SD-SCYB4 | Yanbian, Sichuan | 101.54 | 26.96 | S.D |
| SM-JXDY1 | Dayu, Jiangxi | 114.35 | 25.41 | S.M |  | SD-SCYB5 | Yanbian, Sichuan | 101.42 | 26.93 | S.D |
| SM-JXDY2 | Dayu, Jiangxi | 114.35 | 25.41 | S.M |  | SD-SCYB6 | Yanbian, Sichuan | 101.50 | 26.91 | S.D |
| SM-JXDY3 | Dayu, Jiangxi | 114.35 | 25.41 | S.M |  | SD-SCJY1 | Jinyan, Sichuan | 104.06 | 29.65 | S.D |
| SM-JXHNP1 | Chongyi, Jiangxi | 114.30 | 25.48 | S.M |  | SD-SCLS1 | Leshan, Sichuan | 103.94 | 29.74 | S.D |
| SM-JXCY1 | Chongyi, Jiangxi | 114.31 | 25.68 | S.M |  | SD-SCYQ2 | Yuquan, Sichuan | 105.00 | 31.48 | S.D |
| SM-JXCY4 | Chongyi, Jiangxi | 114.09 | 25.70 | S.M |  | SD-SCYQ3 | Yuquan, Sichuan | 105.00 | 31.48 | S.D |
| SM-JXSY1 | Shangyou, Jiangxi | 114.47 | 25.75 | S.M |  | SD-SCYQ4 | Yuquan, Sichuan | 105.00 | 31.48 | S.D |
| SM-JXSY2 | Shangyou, Jiangxi | 114.45 | 25.73 | S.M |  | SD-SCHM2 | Jianyang, Sichuan | 104.55 | 30.97 | S.D |
| SM-JXLN1 | Longnan, Jiangxi | 114.63 | 24.63 | S.M |  | SD-SCCD2 | Jianyang, Sichuan | 104.38 | 30.55 | S.D |
| SM-JXLN2 | Longnan, Jiangxi | 114.81 | 24.70 | S.M |  | SD-SCCD3 | Jianyang, Sichuan | 104.38 | 30.55 | S.D |
| SM-JXLN3 | Longnan, Jiangxi | 114.85 | 24.90 | S.M |  | SD-SCCD5 | Jianyang, Sichuan | 104.37 | 30.57 | S.D |
| SM-JXLN4 | Longnan, Jiangxi | 114.78 | 24.58 | S.M |  | SD-YNSM4 | Songming, Yunnan | 103.03 | 25.35 | S.D |
| SM-JXLN5 | Longnan, Jiangxi | 114.79 | 24.91 | S.M |  | SD-YNSM6 | Songming, Yunnan | 103.04 | 25.35 | S.D |
| SM-JXLN6 | Longnan, Jiangxi | 114.87 | 24.88 | S.M |  | SD-YNSM7 | Songming, Yunnan | 103.04 | 25.35 | S.D |
| SM-JXDN1 | Dingnan, Jiangxi | 114.89 | 24.67 | S.M |  | SD-YNLF1 | Lufeng, Yunnan | 102.03 | 25.15 | S.D |
| SM-JXDN2 | Dingnan, Jiangxi | 114.89 | 24.67 | S.M |  | SD-YNLF3 | Lufeng, Yunnan | 102.03 | 25.15 | S.D |
| SM-JXDN3 | Dingnan, Jiangxi | 114.89 | 24.67 | S.M |  | SD-YNLF5 | Lufeng, Yunnan | 102.04 | 25.15 | S.D |
| SM-JXDN4 | Dingnan, Jiangxi | 114.87 | 24.64 | S.M |  | SD-YNLF6 | Lufeng, Yunnan | 102.04 | 25.15 | S.D |
| SM-HNJZZ1 | Changsha, Hunan | 112.96 | 28.21 | S.M |  | SD-YNHZ1 | Huize, Yunnan | 103.83 | 26.48 | S.D |
| SM-HNJZZ2 | Changsha, Hunan | 112.96 | 28.21 | S.M |  | SD-YNLQ1 | Luquan, Yunnan | 102.04 | 25.55 | S.D |
| SM-HNJZZ5 | Changsha, Hunan | 112.63 | 28.21 | S.M |  | SD-YNLQ2 | Luquan, Yunnan | 102.04 | 25.55 | S.D |
| SM-HNJZZT1 | Changsha, Hunan | 112.95 | 28.17 | S.M |  | SD-YNAN1 | Anning, Yunnan | 102.47 | 24.96 | S.D |
| SM-HNCS1 | Changsha, Hunan | 113.00 | 28.13 | S.M |  | SD-YNAN2 | Anning, Yunnan | 102.47 | 24.96 | S.D |
| SM-HNCS3 | Changsha, Hunan | 113.02 | 28.10 | S.M |  | SD-YNAN3 | Anning, Yunnan | 102.47 | 24.96 | S.D |
| SM-HNHS1 | Hengyang, Hunan | 112.73 | 27.27 | S.M |  | SD-YNAN4 | Anning, Yunnan | 102.47 | 24.96 | S.D |
| SM-GZLD1 | Luodian, Guizhou | 106.87 | 25.54 | S.M |  | SM-YNSL1 | Shilin, Yunnan | 103.33 | 24.82 | S.M |
| SM-GZLD2 | Luodian, Guizhou | 106.88 | 25.54 | S.M |  | SD-YNSL2 | Shilin, Yunnan | 103.33 | 24.82 | S.D |
| SM-GZGL1 | Guanling, Guizhou | 105.63 | 25.69 | S.M |  | SD-YNSL3 | Shilin, Yunnan | 103.33 | 24.82 | S.D |
| SM-GZGL2 | Guanling, Guizhou | 105.64 | 25.69 | S.M |  | SD-YNSL4 | Shilin, Yunnan | 103.33 | 24.82 | S.D |
| SM-GZZF1 | Zhenfeng, Guizhou | 105.88 | 25.20 | S.M |  | SD-YNSL8 | Shilin, Yunnan | 103.33 | 24.82 | S.D |
| SM-GZZF2 | Zhenfeng, Guizhou | 105.80 | 25.19 | S.M |  | SR-YNSP1 | Shiping, Yunnan | 102.63 | 23.67 | S.R.V |
| SM-GZZF6 | Zhenfeng, Guizhou | 105.84 | 25.18 | S.M |  | SR-YNSP2 | Shiping, Yunnan | 102.68 | 23.67 | S.R.V |
| SM-GZZF7 | Zhenfeng, Guizhou | 105.84 | 25.18 | S.M |  | SR-YNSP3 | Shiping, Yunnan | 102.68 | 23.67 | S.R.V |
| SM-GZCH2 | Ceheng, Guizhou | 105.75 | 25.00 | S.M |  | SM-YNGM1 | Gengma, Yunnan | 99.15 | 23.62 | S.M |
| SM-GZCH3 | Ceheng, Guizhou | 105.75 | 25.00 | S.M |  | SR-YNGM3 | Gengma, Yunnan | 99.15 | 23.62 | S.R |
| SM-GZCH4 | Ceheng, Guizhou | 105.75 | 25.00 | S.M |  | SR-YNGM4 | Gengma, Yunnan | 99.15 | 23.62 | S.R |
| SM-GZDJ1 | Dejiang, Guizhou | 108.31 | 28.32 | S.M |  | SR-YNJS1 | Jianshui, Yunnan | 102.83 | 23.82 | S.R.V |
| SM-GZDJ3 | Dejiang, Guizhou | 108.31 | 28.32 | S.M |  | SD-YNJS4 | Jianshui, Yunnan | 102.83 | 23.84 | S.D |
| SM-GZDJ4 | Dejiang, Guizhou | 108.28 | 28.35 | S.M |  | SR-YNLC1 | Lincang, Yunnan | 100.10 | 23.90 | S.R |
| SM-GZDJ5 | Dejiang, Guizhou | 108.28 | 28.35 | S.M |  | SR-YNPW1 | Puwen, Yunnan | 101.04 | 22.46 | S.R |
| SM-GZZF8 | Zhenfeng, Guizhou | 105.80 | 25.19 | S.M |  |  |  |  |  |  |

Note: S.M indicated *S. mukorossi*, S.D indicated *S. delavayi*, S.R indicated *S. rarak*, S.R.V indicated *S. rarak var*.

Table S2

| Name | Location | Latitude | Longtitude | Species |  | Name | Location | Latitude | Longtitude | Species |
| --- | --- | --- | --- | --- | --- | --- | --- | --- | --- | --- |
| SM-JSHGS1 | Lianyungang, Jiangsu | 119.29 | 34.64 | S.M |  | SM-FJYK3 | Shunchang, Fujian | 117.91 | 26.80 | S.M |
| SM-JSNJ2 | Nanjing, Jiangsu | 118.16 | 32.16 | S.M |  | SM-FJZS1 | Sanming, Fujian | 117.43 | 26.18 | S.M |
| SM-NBYD3 | Yidu, Hubei | 111.37 | 30.18 | S.M |  | SM-FJNH1 | Ninghua, Fujian | 116.74 | 26.27 | S.M |
| SM-CQJY1 | Chongqin | 106.40 | 29.84 | S.M |  | SM-FJNH2 | Ninghua, Fujian | 116.73 | 26.27 | S.M |
| SM-ZJWZ3 | Wenzhou, Zhejiang | 120.66 | 28.02 | S.M |  | SM-FJJN1 | Jianning, Fujian | 112.72 | 27.27 | S.M |
| SM-ZJNB1 | Ningbo, Zhejiang | 121.86 | 29.96 | S.M |  | SM-FJJK1 | Jianning, Fujian | 116.80 | 26.66 | S.M |
| SM-ZJNB2 | Ningbo, Zhejiang | 121.86 | 29.96 | S.M |  | SM-FJJN8 | Jianning, Fujian | 112.72 | 27.27 | S.M |
| SM-ZJLA1 | Hangzhou, Zhejiang | 118.90 | 30.11 | S.M |  | SM-FJHB1 | Jianning, Fujian | 112.72 | 27.27 | S.M |
| SM-ZJHZ1 | Hangzhou, Zhejiang | 120.12 | 30.26 | S.M |  | SM-FJSC1 | Shunchang, Fujian | 116.80 | 26.66 | S.M |
| SM-JXLS1 | Jiujiang, Jiangxi | 115.89 | 29.49 | S.M |  | SM-GDXY1 | Xinyi, Guangdong | 111.61 | 22.49 | S.M |
| SM-JXLS2 | Jiujiang, Jiangxi | 115.89 | 29.49 | S.M |  | SM-GDSG1 | Shaoguan, Guangdong | 113.75 | 25.04 | S.M |
| SM-JXLS4 | Jiujiang, Jiangxi | 115.98 | 29.55 | S.M |  | SM-GDYF18 | Yunfu, Guangdong | 111.98 | 22.75 | S.M |
| SM-JXJN4 | Nanchang, Jiangxi | 115.83 | 28.77 | S.M |  | SM-GDXF1 | Yunfu, Guangdong | 111.96 | 39.86 | S.M |
| SM-JXJN6 | Nanchang, Jiangxi | 115.83 | 28.77 | S.M |  | SM-GDGZ1 | Guangzhou, Guangdong | 113.29 | 23.18 | S.M |
| SM-JXAF1 | Anfu, Jiangxi | 114.58 | 27.39 | S.M |  | SM-GXGL2 | Guilin, Guangxi | 110.36 | 25.31 | S.M |
| SM-JXAF2 | Anfu, Jiangxi | 114.58 | 27.39 | S.M |  | SM-GXGL4 | Guilin, Guangxi | 109.66 | 24.95 | S.M |
| SM-JXFY1 | Fenyi, Jiangxi | 114.74 | 27.79 | S.M |  | SM-GXNN1 | Nanning, Guangxi | 108.49 | 22.75 | S.M |
| SM-JXDY3 | Dayu, Jiangxi | 114.35 | 25.41 | S.M |  | SM-GXLZ1 | Liuzhou, Guangxi | 109.41 | 24.44 | S.M |
| SM-JXCY1 | Chongyi, Jiangxi | 114.31 | 25.68 | S.M |  | SM-HNHK1 | Haikou, Hainan | 110.19 | 19.57 | S.M |
| SM-JXSY1 | Shangyou, Jiangxi | 114.47 | 25.75 | S.M |  | SM-HNTC1 | Tunchang, Hainan | 110.01 | 19.12 | S.M |
| SM-JXLN3 | Longnan, Jiangxi | 114.85 | 24.90 | S.M |  | SM-YN1 | Yuenan | 106.69 | 21.70 | S.M |
| SM-JXDN2 | Dingnan, Jiangxi | 114.89 | 24.67 | S.M |  | SD-SCSF1 | Jianyang, Sichuan | 104.82 | 30.50 | S.D |
| SM-HNJZZT1 | Changsha, Hunan | 112.95 | 28.17 | S.M |  | SD-SCJS1 | Chengdu, Sichuan | 103.80 | 30.38 | S.D |
| SM-HNCS1 | Changsha, Hunan | 113.00 | 28.13 | S.M |  | SD-SCLS1 | Leshan, Sichuan | 103.94 | 29.74 | S.D |
| SM-HNCS3 | Changsha, Hunan | 113.02 | 28.10 | S.M |  | SD-SCYQ2 | Yuquan, Sichuan | 105.00 | 31.48 | S.D |
| SM-GZGL2 | Guanling, Guizhou | 105.64 | 25.69 | S.M |  | SD-YNHZ1 | Huize, Yunnan | 103.83 | 26.48 | S.D |
| SM-GZZF1 | Zhenfeng, Guizhou | 105.88 | 25.20 | S.M |  | SM-YNSL1 | Shilin, Yunnan | 103.33 | 24.82 | S.D |
| SM-GZZF6 | Zhenfeng, Guizhou | 105.84 | 25.18 | S.M |  | SD-YNSL3 | Shilin, Yunnan | 103.33 | 24.82 | S.D |
| SM-GZDJ1 | Dejiang, Guizhou | 108.31 | 28.32 | S.M |  | SD-YNSL8 | Shilin, Yunnan | 103.33 | 24.82 | S.D |
| SM-GZDJ4 | Dejiang, Guizhou | 108.28 | 28.35 | S.M |  | SR-YNSP2 | Shiping, Yunnan | 102.68 | 23.67 | S.R.V |
| SM-GZCH1 | Ceheng, Guizhou | 105.76 | 24.99 | S.M |  | SR-YNJS4 | Jianshui, Yunnan | 102.83 | 23.84 | S.D |
| SM-FJLS4 | Shunchang, Fujian | 117.81 | 26.79 | S.M |  | SR-YNPW1 | Puwen, Yunnan | 101.04 | 22.46 | S.R |

Note: S.M indicated *S. mukorossi*, S.D indicated *S. delavayi*, S.R indicated *S. rarak*, S.R.V indicated *S. rarak var*.
